# Long-term National Trends in Pediatric Oral Antibiotic Prescriptions in Japan, 2011–2022

**DOI:** 10.31662/jmaj.2026-0031

**Published:** 2026-08-07

**Authors:** Yusuke Okubo, Kazuhiro Uda, Noriko Iwamoto, Isao Miyairi

**Affiliations:** 1 Department of Social Medicine, National Center for Child Health and Development, Tokyo, Japan; 2 Department of Pediatric Regional Healthcare, Faculty of Medicine, Dentistry, and Pharmaceutical Science, Okayama University, Okayama, Japan; 3 Disease Control and Prevention Center, National Center for Global Health and Medicine, Japan Institute for Health Security, Tokyo, Japan; 4 Department of Pediatrics, Hamamatsu University School of Medicine, Shizuoka, Japan; 5 Department of Microbiology, Immunology, and Biochemistry, University of Tennessee Health Science Center, Memphis, TN, USA

**Keywords:** pediatric antibiotic prescriptions, National Database (Japan), antimicrobial stewardship, interrupted time series

## Abstract

**Introduction:**

Antimicrobial resistance is a major global health challenge, prompting Japan to implement a National Action Plan in 2016. This study examined long-term national trends from 2011 to 2022 to evaluate the impact of national health policy and identify priority areas for future antimicrobial stewardship efforts.

**Methods:**

We conducted time-series analyses of oral antibiotic prescriptions among children aged 0-19 years in Japan using 408 million outpatient claims from the National Database between fiscal years 2011 and 2022. Antibiotic use was measured as days of therapy per 1,000 pediatric inhabitants per day (DOTs/PID). Trends and relative changes were evaluated using multivariable Poisson regression, with additional analyses stratified by age and prefecture.

**Results:**

Total antibiotic prescriptions decreased by 58.6% between 2011 and 2022 (from 33.4 to 13.8 DOTs/PID), with a marked slope change (–8.2%) following the implementation of the National Action Plan in 2016 and a substantial level drop (–36.4%) during the coronavirus disease 2019 pandemic. We also observed marked reductions in macrolides and cephalosporins but a 23.9% increase in tetracyclines. Reductions were limited among adolescents aged 15-19 years, and tetracycline use increased particularly after 2017. All prefectures showed declines in antibiotic use (38%-67%), although quinolone prescriptions varied widely, and tetracycline use increased in most prefectures.

**Conclusion:**

Pediatric antibiotic prescriptions in Japan decreased substantially between 2011 and 2022, particularly for macrolides and cephalosporins, while tetracycline use increased among adolescents. These long-term national trends provide important implications for the need for sustained, age-specific interventions in the post-pandemic era.

## Introduction

The overuse of antibiotics remains an unresolved challenge in global and regional health ^(1), (2), (3), (4), (5)^. The number of deaths owing to antimicrobial resistance (AMR) worldwide was estimated at 700,000 in 2010 ^(6)^. More recent analyses suggest that AMR was directly responsible for 1.14 million deaths in 2021 and is projected to cause around 1.9 million deaths per year by 2050 ^(7)^. Ensuring appropriate access to antibiotics while avoiding unnecessary use has therefore become a global health priority, as the overuse and misuse of antibiotics remain key drivers of AMR, although the causes of antibiotic resistance are complex and involve human behaviors at many levels of society.

To tackle this challenge, the Ministry of Health, Labour and Welfare in Japan established its first National Action Plan on AMR in 2016 ^(8), (9)^. This health policy aimed to reduce total antibiotic use by 33% by 2020, compared with 2013, through a multifaceted strategy including improving public awareness, strengthening surveillance, and promoting antimicrobial stewardship at medical institutions ^(9)^. Consequently, total antibiotic use declined by 28.9% from 2013 to 2020 ^(10)^. Recently, the National Action Plan on AMR was revised to set new targets, including a further 15% reduction in total antibiotic use from 2020 to 2027 ^(8), (9)^.

However, nationwide studies on pediatric antibiotic use in Japan have been relatively scarce. Previous national analyses covered data up to 2018 ^(4), (11)^, leaving uncertainty regarding recent trends, including the impact of the coronavirus disease 2019 (COVID-19) pandemic, age-specific patterns, and regional variation, and progress toward policy targets. Therefore, this study examined long-term national trends in pediatric antibiotic prescriptions in Japan from 2011 to 2022 using the National Database to identify priority areas for strengthening future antimicrobial stewardship policies.

## Materials and Methods

### Study design, data source, and study population

We conducted time-series analyses to examine trends in oral antimicrobial use among children aged 0-19 years in Japan, using data from the National Database (NDB) between April 2011 and March 2023. The study protocol was approved by the Institutional Review Board (IRB) of the National Center for Child Health and Development, Japan (IRB number, 2023-052). The requirement for informed consent was waived because the analyses were based on anonymized secondary administrative data.

The Ministry of Health, Labour and Welfare approved our data request and extracted the relevant data from the NDB. Owing to Japan’s universal healthcare system, the NDB contains almost all outpatient claims data (>99% coverage), corresponding to approximately one billion data elements annually ^(12)^. All patient- and medical facility-level identifiers were anonymized before being transferred to the NDB for research use. We were granted access to essential variables, including patient age in years, diagnoses, procedures, prescriptions, and the prefectures in which each medical facility was located. For the present analysis, antibiotic prescription data were extracted irrespective of diagnoses or procedures.

For this study, we used 408,437,744 records of children aged 0-19 years who visited medical facilities and received antibiotic prescriptions between April 2011 and March 2023, corresponding to fiscal years 2011-2022. The age range was defined in accordance with commonly used categorizations in national data in Japan, although definitions of pediatric populations vary across studies. For each outpatient visit, the days of therapy were calculated based on information recorded in the prescription claims.

### Measure of antibiotic use and types of antibiotics

The prescription data on oral antibiotics were classified according to the Anatomical Therapeutic Chemical system. In Japan, antibiotics are available only with a physician’s prescription and are dispensed by pharmacies. Antimicrobials for systemic use were recoded as J01 and further categorized into 11 subgroups, in accordance with previous studies (Supplemental Table 1) ^(3), (4), (11), (13), (14), (15)^. Parenteral and topical antibiotics, as well as antivirals, anti-tuberculosis, antifungals, and antiparasitic agents, were not included in the study.

The primary outcome metric was days of therapy (DOTs) per 1,000 pediatric inhabitants/day (DOTs/PID). Because antibiotic dosing in children varies substantially according to body weight and age, defined daily doses, which are based on standard adult dosing assumptions, are not appropriate for pediatric populations; therefore, DOT has been widely used as a more suitable metric for evaluating antibiotic use in children. DOTs/PID was derived from the total DOTs calculated from prescription claims and the corresponding pediatric population (age 0-19 years) in each of 47 prefectures. Denominators for the pediatric population by prefecture and age group were obtained from the national census in 2015 and 2020. For the other years, the population counts were estimated using population estimates available in 5-year age strata, which required interpolation to obtain single-year age distributions. Age-specific distributions were therefore derived by applying Sprague’s multipliers, a standard demographic method for interpolating single-year age groups ^(16)^.

### Statistical analysis

First, antibiotic prescriptions expressed as DOTs/PID were assessed for total antibiotics and 11 subgroups from fiscal years 2011 to 2022. Then, relative changes in antibiotic prescriptions were assessed using multivariable Poisson regression models, with fiscal year 2011 as the reference year ^(17)^. We used the following multivariable Poisson regression models with robust variance estimates:


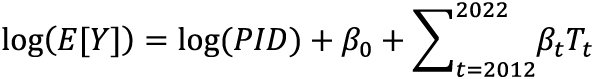


, where Y denotes the number of DOTs for antibiotics, log(PID) is the offset term representing 1,000 person-days for pediatric inhabitants, *T_t_* indicates time-indicator variables for each fiscal year (t). The exponentiated coefficients (*β_t_*) represent the relative changes in antibiotic prescriptions compared with the baseline year (2011), which were effect estimates of interest.

Second, because the pediatric population structure varied across fiscal years, we performed direct standardization using the 2020 pediatric population as the reference and repeated the primary analyses ^(18)^. The standardized rate (DOTs/PID) was calculated using the following formula:


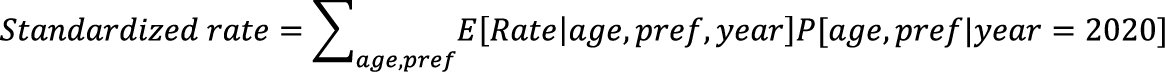


Third, we stratified the data by age group and prefecture and analyzed antibiotic use from fiscal years 2011 to 2022. Fourth, the access rate (Supplemental Table 1) was calculated by dividing the DOTs of access antibiotics by the DOTs of all antibiotics from 2011 to 2022. The distribution of the access rate was further assessed by age group and prefecture.

Fifth, we conducted interrupted time-series (ITS) analyses to evaluate temporal trends in oral antimicrobial use and access rates from 2011 to 2022. The ITS analysis incorporated a linear spline for 2011-2019 with knots at 2013 and 2016, and a segmented linear regression model from 2020 onward to evaluate changes in level and trend associated with the COVID-19 pandemic. We did not include a knot in 2013 in the access rate model because it did not meaningfully improve the model fit. All data were analyzed using Stata/MP software version 19.5 (StataCorp, TX, USA).

## Results

### National trends in antibiotic prescription from fiscal years 2011 to 2022

Figure 1 presents the national trends in antibiotic prescriptions from 2011 to 2022. Total antibiotic prescriptions decreased from 33.4 DOTs/PID in 2011 to 13.8 DOTs/PID in 2022, corresponding to a 58.6% relative reduction (95%CI, 56.0% to 61.1%). Similarly, we observed a 68.5% reduction in macrolides (from 13.3 to 4.19 DOTs/PID), a 67.8% reduction in cephalosporins (from 12.4 to 3.98 DOTs/PID), and a 31.1% reduction in quinolones (from 1.45 to 1.00 DOTs/PID; Supplemental Table 2). In contrast, tetracycline prescription increased by 23.9% from 1.39 DOTs/PID in 2011 to 1.72 DOTs/PID in 2022 (Supplemental Figure 1).

**Figure 1.**
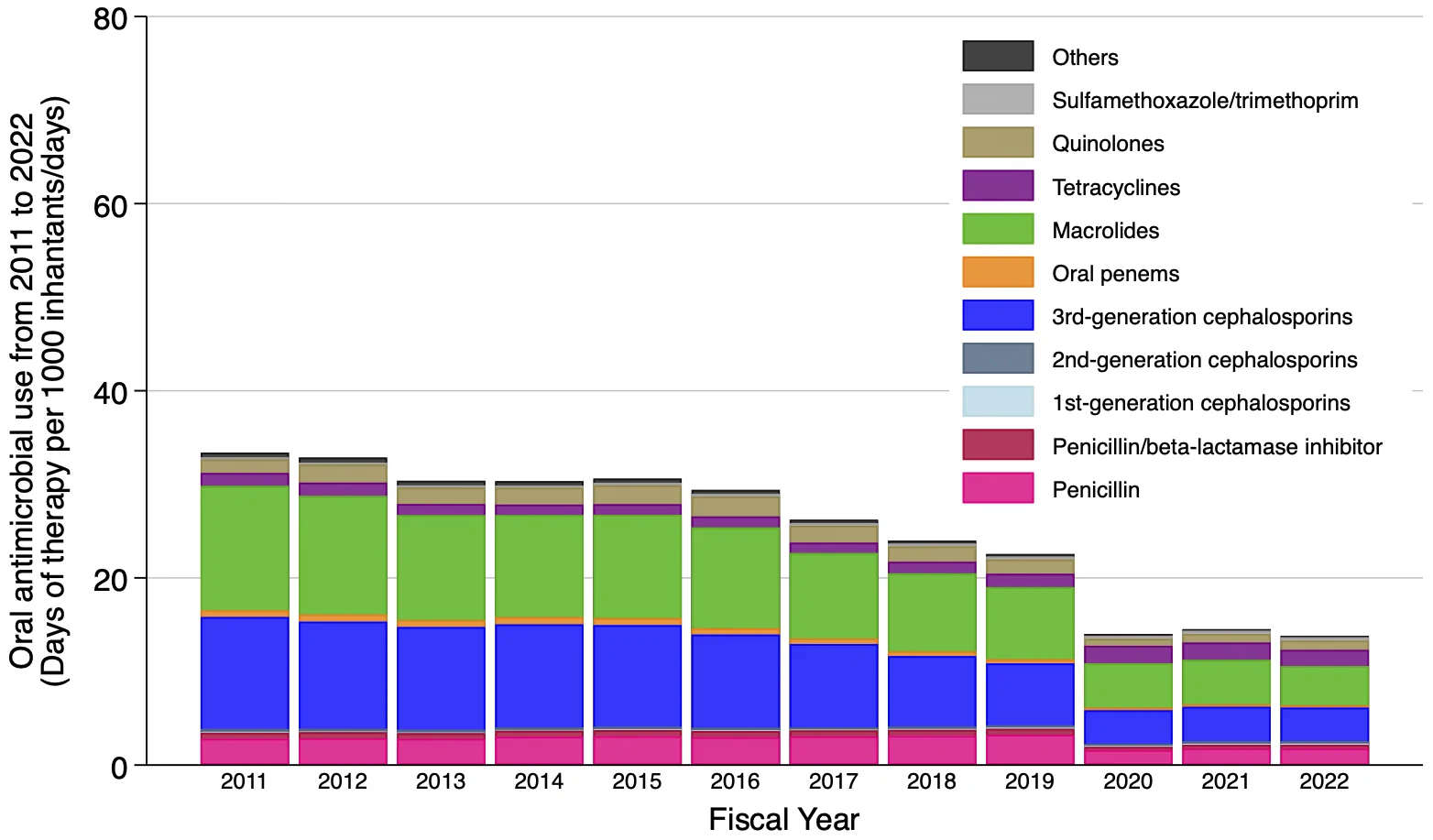
Annual trends in oral antibiotic use among children in Japan, fiscal years 2011-2022.

To account for changes in population structure, we applied the standardization formula. The results were largely unchanged, as shown in Supplemental Table 3.

### National trends in antibiotic prescriptions by age group and prefectures

Figure 2 and Supplemental Table 4 show age-specific trends in antibiotic prescriptions. Reductions in antibiotic use were greater in younger children and gradually decreased with age, whereas the changes were modest among adolescents aged 15-19 years. The highest prescription rates were observed in children aged 1-5 years; in this age group, prescriptions decreased by approximately 57%-73%. In contrast, reductions among those aged 15-19 years were limited, ranging from 3% to 19%. Notably, the decline in quinolone prescriptions was minimal among children aged ≤3 years, and tetracycline use increased among adolescents aged ≥13 years, particularly after 2017.

**Figure 2.**
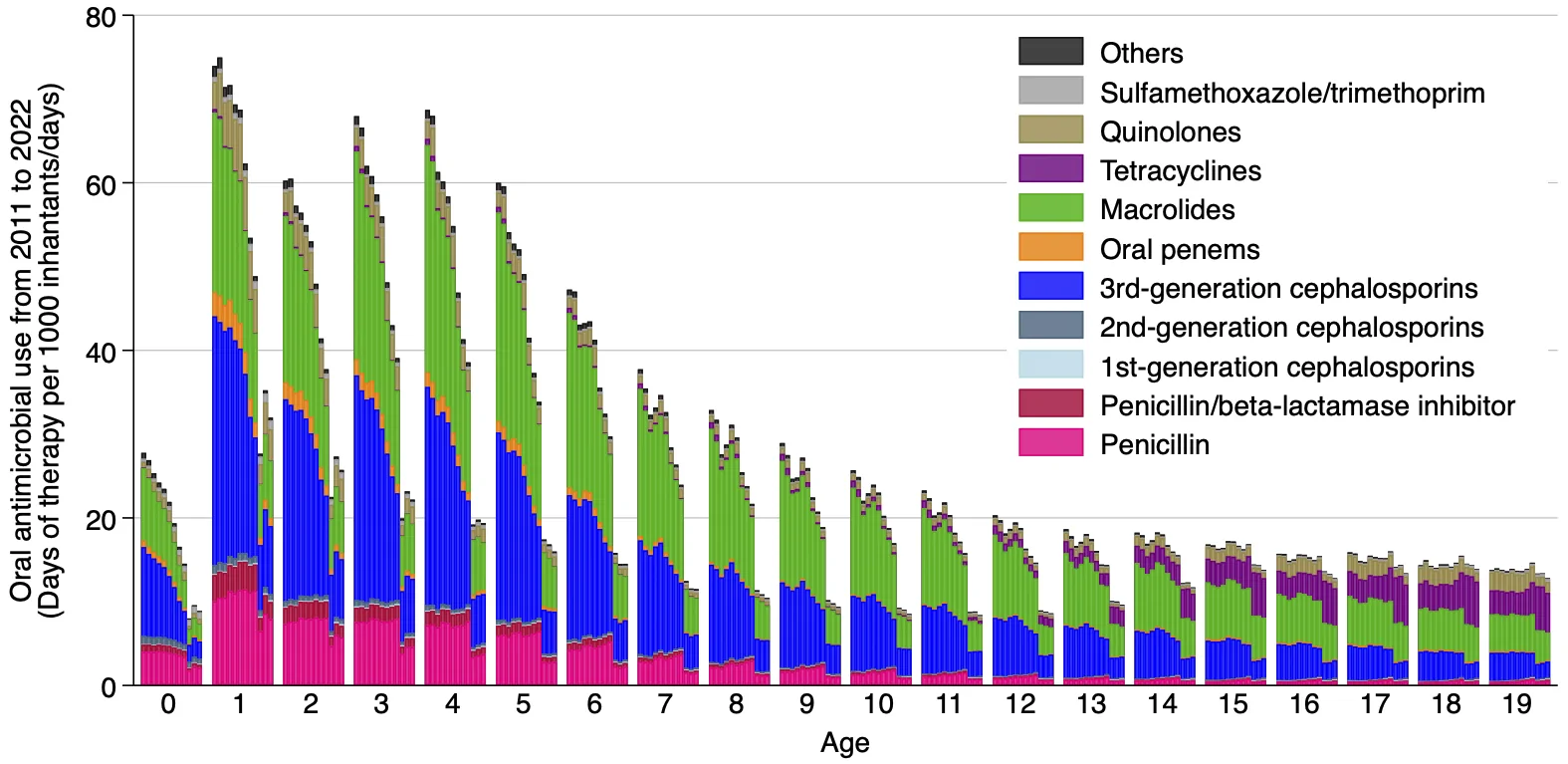
Annual trends in oral antibiotic use by age group among children in Japan, fiscal years 2011-2022.

Figure 3 and Supplemental Table 5-7 show prefecture-specific trends in antibiotic prescriptions. Overall, antibiotic use markedly decreased in all prefectures, with reductions ranging from 38% to 67%. Prefectures with approximately 30-40 DOTs/PID in 2011 showed reductions to around 20 DOTs/PID by 2019 and to around 15 DOTs/PID by 2022. In contrast, quinolone prescriptions varied substantially across prefectures, ranging from a 48% relative increase to a 73% relative reduction. In most prefectures (41/47), tetracycline use increased, with the largest relative increase reaching 150%.

**Figure 3.**
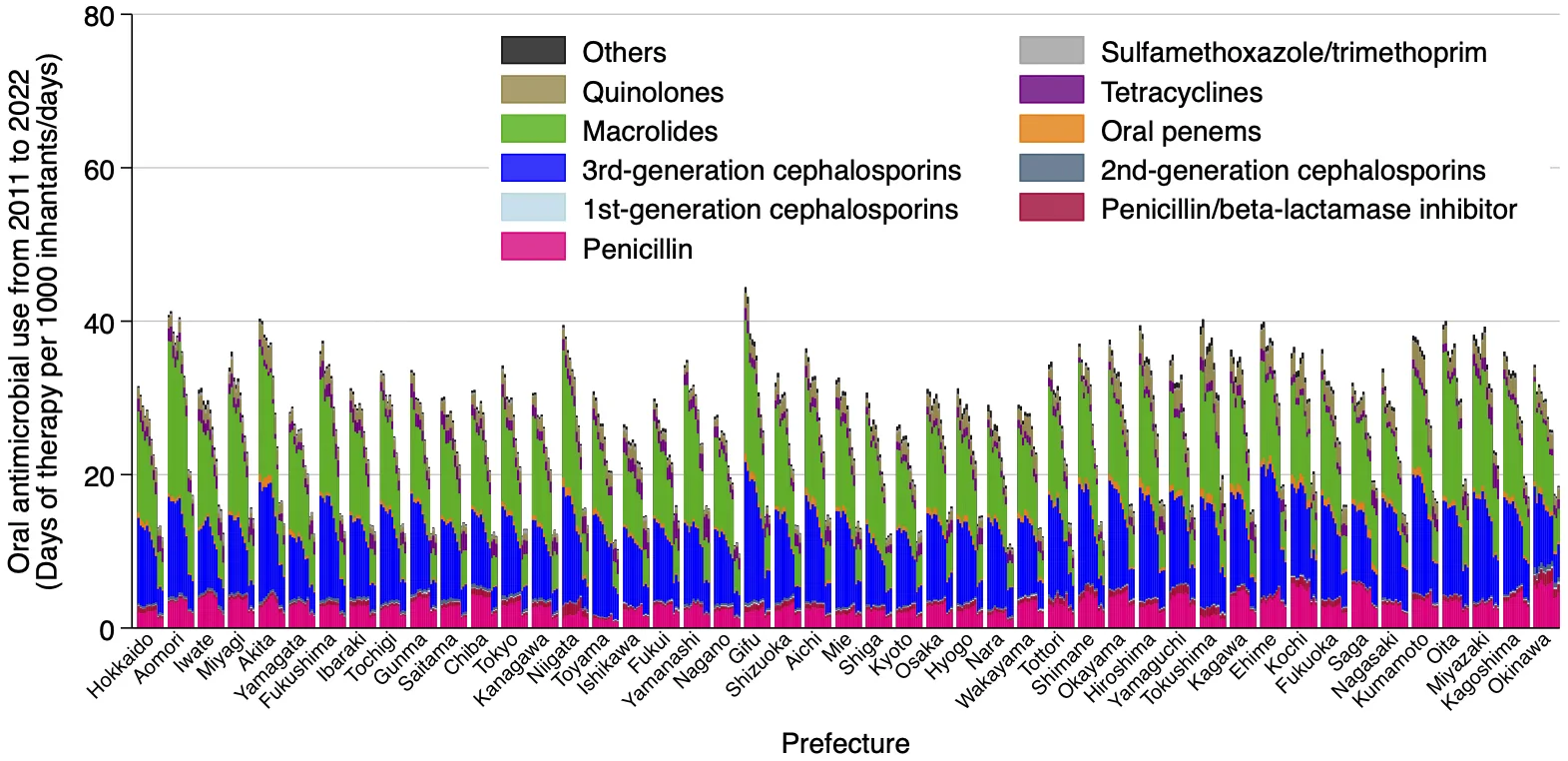
Geographic variation in pediatric oral antibiotic use across Japan, fiscal years 2011-2022.

### Access rates from fiscal years 2011 to 2022

The access rate showed a steady increase from 11.2% in 2011 to 19.0% in 2019, with a transient decrease observed in 2020 (16.8%) during the COVID-19 pandemic, and then recovered to 19.0% by 2022 (Supplemental Figure 2). Access rates increased across all age groups from 2011 to 2022, with roughly a twofold relative increase overall, whereas the magnitude of absolute increase was smaller among older children. Across prefectures, access rates increased by roughly 1.5-fold from 2011 to 2022. The absolute increase varied geographically, with Okinawa Island showing the most marked rise (from 20.7% to 34.1%).

### Interrupted time-series analyses for oral antibiotic use and access rates

Oral antimicrobial use showed a slight decline until 2013 (4.9% decrease [95% CI, 1.6%-8.0%]), followed by a stable period through 2015 (Figure 4). A marked downward trend began after the implementation of the National Action Plan on Antimicrobial Resistance in 2016 (rate ratio, 0.918 [95% CI, 0.883-0.954]), and a substantial level drop (36.4% [95% CI, 32.6%-39.8%]) was observed in 2020 during the COVID-19 pandemic, after which the trend remained stable (Supplemental Table 8).

**Figure 4.**
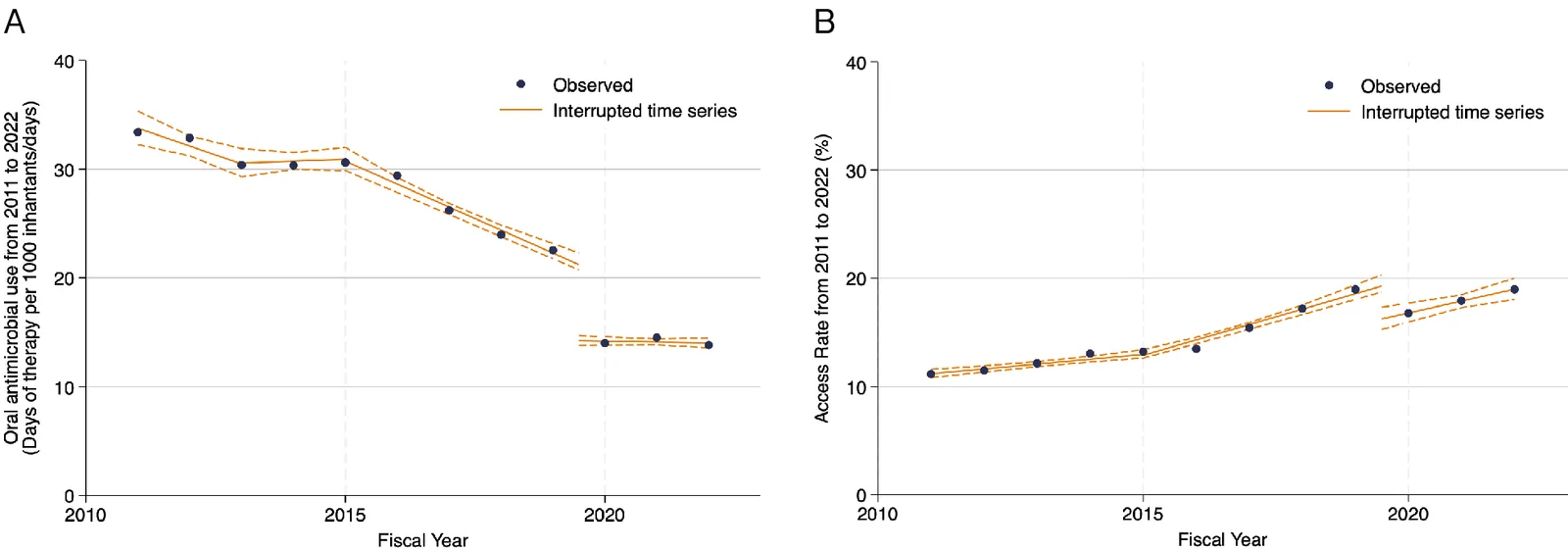
Interrupted time-series analyses of oral antibiotic use and access rate, 2011-2022. Trends in (A) oral antibiotic use and (B) access antibiotic rate in Japan from 2011 to 2022.

Access rates increased steadily from 2011 to 2019 (baseline trend, 3.9% [95% CI, 2.5%-5.2%]), and the upward trend accelerated after the implementation of the National Action Plan on AMR in 2016 (rate ratio, 1.095 [95% CI, 1.081-1.110]). During the COVID-19 pandemic in 2020, access rates dropped by 15.5% (95% CI, 7.2%-23.1%), and a modest increasing trend thereafter (slope, 6.3% [95% CI, 2.2%-10.7%]).

## Discussion

In our national study in Japan, antibiotic prescriptions decreased substantially between 2011 and 2022, with marked reductions in macrolides and cephalosporins. Reductions were largest among children aged ≤10 years, whereas decreases were limited among adolescents aged 15-19 years. All prefectures showed decreases in antibiotic use, although the magnitude varied, and tetracycline use increased in most prefectures.

In high-income countries, pediatric antibiotic prescribing has generally declined, although the magnitude and pattern of reduction vary across countries. For example, outpatient use fell by 29%-49% across four Nordic countries between 2006/2007 and 2016/2017 ^(19)^, and by 43% in Germany between 2010 and 2018 ^(20)^, whereas in the United States, overall prescribing declined between 2000 and 2010 but broad-spectrum prescribing increased, with additional pandemic-related fluctuations thereafter ^(21)^. Our findings extend previous research on pediatric antibiotic use in Japan by demonstrating long-term national trends up to 2022. Our earliest analysis using the NDB from 2013 to 2016 found no reduction in overall antibiotic use, with prescriptions for cephalosporins, macrolides, and quinolones remaining unchanged ^(11)^. The highest use was observed in children aged 1-5 years with respiratory tract infections ^(3)^. Another study, using the NDB from 2011 to 2018, compared antibiotic use before and after the National Action Plan for AMR, showing an 18.7% reduction in overall use (2011-2012 vs 2017-2018) ^(4)^. This reduction was accompanied by marked decreases among children aged ≤8 years but increases among those aged ≥15 years. In the present study, extending the analysis through fiscal year 2022, we found a 58.6% reduction in total antibiotic prescriptions compared with fiscal year 2011, with declines in macrolides (68.5%), cephalosporins (67.8%), and quinolones (31.1%), whereas tetracycline prescriptions increased by 23.9%. The marked reduction in pediatric antibiotic use is likely attributable to multiple factors, including the implementation of several health policies by the Japanese government as well as changes in infectious disease epidemiology and healthcare-seeking behavior during the COVID-19 pandemic.

The National Action Plan on AMR (2016-2020) was implemented in the context of broader antimicrobial stewardship efforts in Japan, including the publication and revision of national guidance, and set a numerical target to reduce total antibiotic use by 33% and the use of oral cephalosporins, macrolides, and fluoroquinolones by 50% by 2020, compared with 2013 levels ^(9)^. These targets likely functioned as clear benchmarks for healthcare providers and may have contributed to changes in prescribing practices. Furthermore, in 2018, a financial incentive system was introduced for pediatricians treating children under 3 years of age, allowing additional reimbursement when parents were informed that antibiotics were unnecessary for conditions such as acute upper respiratory infections and gastroenteritis ^(22)^. This scheme was revised in 2020 to expand eligibility to children up to 6 years of age ^(15)^. In our previous studies, this financial incentive appeared to provide a substantial boost, associated with a more than 20% reduction in antibiotic prescriptions, and its effect was sustained for four years ^(15), (22)^. Notably, in the present study, we also observed a spillover effect, with reductions in antibiotic use among children aged ≥6 years who were not directly targeted by this incentive, possibly reflecting pediatricians’ broader reconsideration of their prescribing practices.

Our findings also have policy implications for future antimicrobial stewardship strategies in Japan. First, the reduction in antibiotic prescriptions was limited among adolescents aged ≥15 years. In Japan, patients in this age group are usually treated by internists rather than pediatricians, and therefore, pediatric-focused policies may not have effectively reached this population. Second, the use of third-generation cephalosporins remains high, highlighting the need for physicians to preferentially select narrower-spectrum antibiotics (e.g., penicillin or first-generation cephalosporin, as first-line agents), with a low access rate overall (far below the World Health Organization target of 60%). Notably, Okinawa showed the highest access rate among prefectures, suggesting that local prescribing practices or antimicrobial stewardship activities may contribute to this regional pattern ^(23)^. Recently, Japan introduced a monitoring and feedback system for antimicrobial use through the 2024 reimbursement revision, which may help promote greater use of access antibiotics across regions. Third, although quinolone prescriptions decreased overall, the decline was less pronounced among younger children, suggesting room for further improvement because most pathogens in this age group can be treated with alternative antibiotics ^(24)^. Finally, tetracycline prescriptions increased markedly among adolescents aged ≥13 years, consistent with our previous findings that dermatology accounted for a large proportion of tetracycline use for acne vulgaris ^(4)^. This trend may be related to the standardization of acne management following the 2017 guideline revision, as well as the recognition of doxycycline use for acne in reimbursement practice in 2018 ^(25)^. Although tetracyclines are also used for Mycoplasma infections, no major nationwide epidemic was observed after 2017, making this explanation less likely for the sustained increase ^(26)^.

This study has several limitations. The claims database used in this study did not fully cover the entire Japanese population, as individuals receiving public assistance were not included, which may have led to an underestimation of antibiotic use. Because the estimates are based on prescription records rather than actual drug intake, they may overestimate true antimicrobial exposure. DOT reflects the duration of antibiotic exposure but does not capture the prescribed dose or total amount of antibiotics used. Because the database does not include clinical information such as vaccination history, symptoms, or laboratory results, we were unable to assess the appropriateness of antibiotic use or to evaluate whether the observed reductions in antibiotic use had any unintended impact on infectious disease outcomes. Additionally, the database lacked background information, such as reasons for visits, which makes it difficult to evaluate shifts in health-seeking behavior. In particular, from 2020 onward, infection prevention measures related to the COVID-19 pandemic were implemented in schools and childcare facilities for three years ^(27), (28), (29)^. Part of the observed reduction in antibiotic prescriptions may reflect these measures (a level drop by 36.4% in the ITS analysis), and a rebound is likely occurring from fiscal year 2023, as such measures have been relaxed. In addition, these changes should be interpreted with caution, as multiple factors may have contributed to the observed trends.

## Conclusions

Pediatric antibiotic prescriptions in Japan decreased substantially between 2011 and 2022, particularly for macrolides and cephalosporins, although tetracycline use increased among adolescents. The reductions were most pronounced among children ≤10 years, while decreases were limited among adolescents ≥15 years, and all prefectures showed consistent but variable declines. These long-term national trends likely reflect the impact of multiple health policies, providing important implications for future antimicrobial stewardship strategies.

## Article Information

### Author Contributions

Yusuke Okubo conceived the study, coordinated data management, performed the analyses, and drafted the manuscript. Kazuhiro Uda, Noriko Iwamoto, and Isao Miyairi contributed to study design and interpretation of the findings, critically revised the manuscript for important intellectual content, and approved the final version. All authors acquired access to the administrative claims data, read and approved the submitted manuscript, and agree to be accountable for all aspects of the work.

### Conflicts of Interest

Kazuhiro Uda belongs to an endowed department supported by a donation from Setouchi City, Okayama, Japan, and declares no conflicts of interest directly related to this study. Isao Miyairi has received honoraria for lectures from the following entities: Shionogi & Co., Ltd.; Takeda Pharmaceutical Company Limited; Daiichi Sankyo Company, Limited; Sanofi; Kyorin Pharmaceutical Co., Ltd.; Miyarisan Pharmaceutical Co., Ltd.; Meiji Seika Pharma Co., Ltd.; and AstraZeneca. All other authors (Yusuke Okubo, Kazuhiro Uda, and Noriko Iwamoto) report no potential conflicts.

### IRB Approval Code and Name of the Institution

The National Center for Child Health and Development, Japan (IRB number, 2023-052)

## Supplement

Supplementary Material
