# Supplementary material for: Long-term National Trends in Pediatric Oral Antibiotic Prescriptions in Japan, 2011–2022
Source: JMA J. 2026 Aug 7;9(5):1156–63. doi: 10.31662/jmaj.2026-0031 (PMC13639659; doi:10.31662/jmaj.2026-0031)

Index: Supplementary Materials Index

Supplemental Table 1. The Classification and List of the Oral Antibiotics. 2
Supplemental Table 2. National Trends in Antibiotic Use among Children Aged 0 to 19 Years. 3
Supplemental Table 3. National Trends in Antibiotic Use among Children Aged 0 to 19 Years, after Age-Standardization to 2020. 4
Supplemental Table 4. Age-Specific Trends in Antibiotic Use from 2011 to 2022. 5
Supplemental Table 5. Prefecture-Specific Trends in Antibiotic Use from 2011 to 2022 (1/3). 6
Supplemental Table 6. Prefecture-Specific Trends in Antibiotic Use from 2011 to 2022 (2/3). 7
Supplemental Table 7. Prefecture-Specific Trends in Antibiotic Use from 2011 to 2022 (3/3). 8
Supplemental Table 8. Interrupted Time-Series Analyses of Oral Antibiotic Use and Access Rate, 2011–2022 9
Supplemental Figure 1. National trends in antibiotic use from 2011 to 2022 stratified by antibiotic types. 10
Supplemental Figure 2. National trends in access rates from 2011 to 2022. 12

Supplemental Table 1. The Classification and List of the Oral Antibiotics.

Classification	Details
Penicillin	Benzylpenicillin benzathine hydrate, ampicillin, bacampicillin hydrochloride, amoxicillin hydrate
Penicillin with beta-lactamase inhibitors	Amoxicillin clavulanate, sultamicillin
First-generation cephalosporin	Cefalexin, cefadroxil, cefatrizine, cefroxadine
Second-generation cephalosporin	Cefuroxime axetil, cefaclor, cefotiam pivoxil
Third-generation cephalosporin	Cefdinir, ceftibuten, cefditoren pivoxil, cefixime, cefteram pivoxil, cefpodoxime proxetil, cefcapene pivoxil
Oral penem	Tebipenem pivoxil, faropenem
Macrolide	Erythromycin, clarithromycin, roxithromycin, azithromycin, josamycin
Tetracycline	Tetracycline, doxycycline, minocycline
Quinolone	Norfloxacin, ofloxacin, levofloxacin, ciprofloxacin, lomefloxacin, tosufloxacin, prulifloxacin, moxifloxacin, garenoxacin, sitafloxacin
Trimethoprim-sulfamethoxazole	Trimethoprim-sulfamethoxazole
Others	Clindamycin, fosfomycin, linezolid, chloramphenicol

***Access antibiotics:** doxycycline, tetracycline, chloramphenicol, ampicillin, amoxicillin, bacampicillin, benzathine benzylpenicillin, amoxicillin and clavulanic acid, sultamicillin, cefalexin, cefadroxil, cefatrizine, cefroxadine, sulfamethizole, sulfamethoxazole, sulfadimethoxine, sulfamethoxazole and trimethoprim, clindamycin

Supplemental Table 2. National Trends in Antibiotic Use among Children Aged 0 to 19 Years.

**Antibiotics**	**Total**				**Macrolides**				**Cephalosporins**			
Year	Rate	RR	95%LL	95%UL	Rate	RR	95%LL	95%UL	Rate	RR	95%LL	95%UL
2011	33.4	1.000			13.3	1.000			12.4	1.000		
2012	32.9	0.984	0.916	1.057	12.7	0.952	0.891	1.019	11.8	0.955	0.879	1.039
2013	30.4	0.910	0.847	0.977	11.2	0.843	0.789	0.900	11.3	0.917	0.844	0.997
2014	30.3	0.909	0.846	0.975	10.9	0.819	0.768	0.874	11.4	0.919	0.847	0.999
2015	30.6	0.917	0.856	0.982	11.0	0.830	0.780	0.884	11.2	0.907	0.837	0.983
2016	29.4	0.881	0.822	0.943	10.8	0.809	0.761	0.861	10.3	0.834	0.770	0.903
2017	26.2	0.785	0.734	0.840	9.14	0.687	0.647	0.729	9.24	0.747	0.691	0.808
2018	24.0	0.718	0.673	0.766	8.33	0.626	0.591	0.664	7.89	0.638	0.591	0.689
2019	22.6	0.675	0.634	0.719	7.71	0.580	0.548	0.613	6.99	0.565	0.525	0.609
2020	14.0	0.419	0.396	0.445	4.75	0.357	0.339	0.377	3.91	0.316	0.294	0.340
2021	14.5	0.435	0.409	0.463	4.74	0.356	0.337	0.376	4.05	0.328	0.305	0.353
2022	13.8	0.414	0.389	0.440	4.19	0.315	0.299	0.333	3.98	0.322	0.299	0.346
%Reduction		58.6	61.1	56.0		68.5	70.1	66.7		67.8	70.1	65.4
**Antibiotics**	**Quinolones**				**Penicillins**				**Tetracyclines**			
Year	Rate	RR	95%LL	95%UL	Rate	RR	95%LL	95%UL	Rate	RR	95%LL	95%UL
2011	1.45	1.000			2.81	1.000			1.39	1.000		
2012	1.92	1.323	1.231	1.423	2.86	1.019	0.897	1.157	1.42	1.025	0.921	1.141
2013	1.76	1.211	1.123	1.307	2.80	0.997	0.878	1.132	1.20	0.864	0.765	0.976
2014	1.79	1.235	1.143	1.333	3.04	1.081	0.953	1.226	1.13	0.811	0.716	0.919
2015	2.02	1.392	1.291	1.500	3.09	1.100	0.972	1.245	1.13	0.818	0.725	0.923
2016	2.13	1.469	1.361	1.587	2.98	1.061	0.937	1.201	1.18	0.849	0.754	0.955
2017	1.82	1.250	1.153	1.355	3.07	1.091	0.965	1.234	1.10	0.793	0.696	0.903
2018	1.65	1.135	1.047	1.229	3.12	1.110	0.983	1.253	1.23	0.887	0.774	1.015
2019	1.53	1.054	0.973	1.141	3.25	1.156	1.025	1.304	1.42	1.021	0.890	1.172
2020	0.76	0.525	0.484	0.569	1.60	0.571	0.506	0.644	1.87	1.346	1.166	1.554
2021	0.91	0.629	0.573	0.692	1.77	0.631	0.555	0.718	1.85	1.336	1.158	1.541
2022	1.00	0.689	0.628	0.756	1.77	0.629	0.554	0.715	1.72	1.239	1.075	1.428
%Reduction		31.1	37.2	24.4		37.1	44.6	28.5		-23.9	-7.5	-42.8

Supplemental Table 3. National Trends in Antibiotic Use among Children Aged 0 to 19 Years, after Age-Standardization to 2020.

**Antibiotics**	**Total**				**Macrolides**				**Cephalosporins**			
Year	Rate	RR	95%LL	95%UL	Rate	RR	95%LL	95%UL	Rate	RR	95%LL	95%UL
2011	33.1	1.000			13.2	1.000			12.2	1.000		
2012	32.6	0.984	0.914	1.059	12.6	0.952	0.888	1.020	11.7	0.957	0.878	1.042
2013	30.1	0.910	0.846	0.979	11.1	0.843	0.788	0.902	11.2	0.919	0.844	1.001
2014	30.1	0.910	0.846	0.978	10.9	0.821	0.769	0.877	11.3	0.923	0.849	1.004
2015	30.4	0.920	0.857	0.987	11.0	0.834	0.782	0.889	11.1	0.913	0.841	0.990
2016	29.2	0.884	0.824	0.948	10.7	0.812	0.762	0.866	10.2	0.840	0.774	0.911
2017	26.1	0.789	0.737	0.845	9.12	0.690	0.649	0.733	9.19	0.754	0.696	0.816
2018	23.9	0.723	0.677	0.772	8.33	0.629	0.593	0.668	7.86	0.645	0.597	0.697
2019	22.5	0.681	0.639	0.726	7.71	0.583	0.550	0.617	6.98	0.572	0.530	0.617
2020	14.0	0.423	0.399	0.449	4.75	0.359	0.340	0.380	3.91	0.321	0.298	0.345
2021	14.6	0.440	0.413	0.469	4.75	0.359	0.340	0.380	4.07	0.334	0.309	0.360
2022	13.9	0.420	0.394	0.448	4.21	0.318	0.301	0.336	4.00	0.328	0.305	0.354
%Reduction		58.0	60.6	55.2		68.2	69.9	66.4		67.2	69.5	64.6

**Antibiotics**	**Quinolones**				**Penicillins**				**Tetracyclines**			
Year	Rate	RR	95%LL	95%UL	Rate	RR	95%LL	95%UL	Rate	RR	95%LL	95%UL
2011	1.45	1.000			2.76	1.000			1.41	1.000		
2012	1.91	1.317	1.223	1.418	2.81	1.019	0.894	1.162	1.43	1.019	0.913	1.136
2013	1.75	1.202	1.113	1.298	2.76	0.998	0.876	1.137	1.21	0.857	0.758	0.970
2014	1.78	1.223	1.131	1.322	3.00	1.085	0.954	1.235	1.13	0.802	0.706	0.910
2015	2.01	1.379	1.278	1.488	3.06	1.107	0.975	1.256	1.13	0.806	0.713	0.910
2016	2.12	1.457	1.348	1.575	2.95	1.068	0.941	1.213	1.18	0.835	0.742	0.941
2017	1.80	1.239	1.143	1.345	3.04	1.102	0.972	1.250	1.10	0.780	0.684	0.889
2018	1.64	1.128	1.041	1.223	3.11	1.125	0.994	1.273	1.23	0.872	0.761	1.000
2019	1.53	1.051	0.970	1.138	3.24	1.174	1.039	1.327	1.41	1.005	0.875	1.155
2020	0.76	0.525	0.484	0.569	1.60	0.581	0.514	0.657	1.87	1.326	1.147	1.532
2021	0.92	0.633	0.576	0.696	1.79	0.648	0.568	0.738	1.85	1.316	1.139	1.521
2022	1.01	0.696	0.634	0.764	1.80	0.651	0.572	0.740	1.71	1.213	1.052	1.400
%Reduction		30.4	36.6	23.6		34.9	42.8	26.0		-21.3	-5.2	-40.0

Supplemental Table 4. Age-Specific Trends in Antibiotic Use from 2011 to 2022.

Age	0	1	2	3	4	5	6	7	8	9
**Year**										
2011	27.8	74.0	60.3	68.0	68.7	60.0	47.3	37.7	32.9	28.9
2012	26.9	75.0	60.5	66.6	68.0	59.6	47.0	35.5	31.7	27.5
2013	25.3	71.4	57.3	62.0	61.3	54.1	43.1	32.3	27.6	24.7
2014	24.2	71.7	56.4	60.8	60.2	52.7	43.2	33.2	28.8	24.8
2015	23.5	69.4	55.0	58.6	58.4	52.1	43.5	34.7	31.1	27.2
2016	21.9	68.7	53.0	56.0	54.8	49.1	41.3	32.6	29.6	25.9
2017	19.4	62.3	48.0	48.1	46.9	41.5	35.5	28.4	25.5	22.4
2018	16.6	53.4	41.4	43.0	41.3	37.3	32.4	26.4	23.8	20.7
2019	14.5	48.8	37.7	39.1	38.6	33.9	29.7	24.0	21.7	18.9
2020	8.00	27.7	22.5	19.9	19.2	17.4	15.8	12.5	11.4	10.2
2021	9.58	35.2	27.3	23.2	19.8	16.8	14.5	11.6	10.9	9.80
2022	8.90	32.0	25.7	22.2	19.4	16.0	14.5	11.5	10.5	9.39
**%Reduction**	**67.9**	**56.8**	**57.3**	**67.4**	**71.8**	**73.4**	**69.3**	**69.5**	**68.2**	**67.6**

Age	10	11	12	13	14	15	16	17	18	19
**Year**										
2011	25.7	23.3	20.3	18.7	18.2	16.8	15.7	15.9	14.4	13.8
2012	24.9	22.3	19.7	17.8	17.9	16.7	15.7	15.7	15.0	14.0
2013	22.0	20.3	18.1	16.7	16.9	16.3	14.9	14.9	14.3	13.8
2014	22.9	20.7	18.7	17.2	17.3	16.4	15.0	14.7	14.1	13.6
2015	24.0	21.9	19.5	18.1	18.3	17.2	15.6	15.5	14.5	13.9
2016	23.0	20.4	18.8	17.5	18.0	17.2	15.6	15.3	14.5	13.7
2017	20.3	18.2	16.3	16.1	16.7	16.7	15.3	15.2	14.2	13.7
2018	18.8	17.2	15.4	14.4	16.0	16.3	15.0	15.2	14.7	13.9
2019	17.0	15.8	14.7	14.4	15.5	16.9	15.4	16.0	15.5	14.7
2020	9.27	8.77	8.96	10.1	12.2	14.4	13.6	14.2	14.5	13.4
2021	9.05	8.83	8.80	10.0	12.3	14.4	13.3	14.4	14.4	13.4
2022	8.55	8.40	8.61	9.65	11.7	13.8	12.8	13.4	13.9	12.8
**%Reduction**	**66.7**	**63.9**	**57.6**	**48.3**	**35.8**	**18.1**	**18.5**	**15.7**	**3.1**	**7.1**

Supplemental Table 5. Prefecture-Specific Trends in Antibiotic Use from 2011 to 2022 (1/3).

Prefecture	Hokkaido	Aomori	Iwate	Miyagi	Akita	Yamagata	Fukushima	Ibaraki	Tochigi	Gunma
**Year**										
2011	31.5	40.8	31.1	34.0	40.3	28.2	36.1	31.3	33.6	33.6
2012	30.4	41.3	31.3	36.0	40.0	28.8	37.4	30.8	33.2	33.2
2013	29.0	38.7	29.6	32.7	38.3	26.6	33.5	29.1	30.4	30.0
2014	27.6	37.2	29.1	31.7	37.8	25.3	34.1	28.6	29.5	29.9
2015	28.4	38.1	29.7	32.5	36.8	25.9	34.4	29.3	30.4	29.4
2016	27.2	40.5	28.5	30.7	37.2	26.0	32.8	28.6	29.1	28.1
2017	24.6	36.1	26.2	27.7	32.9	23.8	28.9	25.7	25.0	24.6
2018	22.6	32.9	23.5	25.1	28.0	21.2	25.9	23.5	22.9	22.2
2019	20.9	30.5	22.0	23.0	23.7	20.1	23.6	22.0	20.8	21.0
2020	13.1	20.7	16.1	14.3	16.4	14.5	14.9	13.4	13.4	12.4
2021	13.3	20.4	15.5	15.7	16.6	15.1	14.6	13.3	13.6	13.1
2022	11.8	17.3	12.4	14.3	13.6	12.3	12.6	12.6	12.3	12.2
**%Reduction**	**62.7**	**57.5**	**60.0**	**58.0**	**66.1**	**56.4**	**65.0**	**59.8**	**63.3**	**63.8**

Prefecture	Saitama	Chiba	Tokyo	Kanagawa	Niigata	Toyama	Ishikawa	Fukui	Yamanashi	Nagano
**Year**										
2011	30.0	31.0	34.2	30.6	39.5	30.9	26.6	29.9	34.2	27.6
2012	30.1	31.0	33.1	30.7	38.0	30.0	26.3	29.3	34.9	27.8
2013	27.8	28.8	30.2	27.8	36.2	27.9	24.4	27.1	31.0	24.8
2014	27.8	28.3	29.8	27.5	35.2	26.7	24.1	25.7	31.2	25.8
2015	28.3	29.6	30.1	27.8	33.7	26.6	24.6	26.0	31.7	25.4
2016	27.1	28.1	27.8	26.2	32.7	24.9	24.0	25.9	30.5	24.5
2017	24.5	24.5	24.7	23.6	27.7	21.8	21.8	22.2	28.5	21.2
2018	23.0	22.3	22.9	22.2	26.4	20.5	21.6	22.6	24.0	19.7
2019	21.3	20.6	21.1	20.7	24.0	20.5	20.9	21.6	24.1	18.9
2020	13.3	12.1	12.0	12.1	15.5	11.2	14.5	14.9	15.4	10.8
2021	13.8	12.5	12.9	12.8	15.7	11.5	14.7	16.0	16.0	11.1
2022	13.5	12.0	12.9	12.3	13.4	10.3	13.0	14.0	15.6	9.7
**%Reduction**	**55.0**	**61.3**	**62.3**	**60.0**	**66.0**	**66.7**	**51.0**	**53.3**	**54.4**	**64.9**

Supplemental Table 6. Prefecture-Specific Trends in Antibiotic Use from 2011 to 2022 (2/3).

Prefecture	Gifu	Shizuoka	Aichi	Mie	Shiga	Kyoto	Osaka	Hyogo	Nara	Wakayama
**Year**										
2011	44.4	32.0	36.5	32.3	30.7	26.3	31.2	31.2	29.1	29.1
2012	43.2	33.2	35.3	32.7	29.3	26.5	30.8	29.9	28.3	28.9
2013	38.4	29.8	31.9	30.2	26.2	24.6	29.0	28.0	25.8	27.0
2014	37.6	30.5	32.7	30.9	27.2	25.0	29.9	28.6	26.6	28.2
2015	37.3	30.7	32.8	30.8	26.5	25.1	30.5	29.2	26.4	28.0
2016	35.5	28.6	31.2	29.0	24.8	24.1	29.3	27.6	25.0	28.0
2017	30.7	24.1	27.4	26.0	22.6	21.2	27.2	25.1	22.2	26.3
2018	27.8	21.3	25.2	23.6	20.4	19.4	25.1	23.1	19.8	23.7
2019	25.8	20.7	23.0	22.1	18.8	18.7	23.7	21.8	18.5	22.9
2020	14.6	13.4	14.1	13.2	11.7	11.3	14.1	13.5	10.5	13.0
2021	15.9	13.4	14.8	13.9	12.0	12.5	15.1	14.6	11.0	13.1
2022	14.6	12.4	14.3	13.0	12.3	12.7	15.8	14.7	10.5	11.9
**%Reduction**	**67.1**	**61.3**	**60.7**	**59.7**	**59.9**	**51.5**	**49.2**	**53.1**	**64.0**	**59.0**

Prefecture	Tottori	Shimane	Okayama	Hiroshima	Yamaguchi	Tokushima	Kagawa	Ehime	Kochi	Fukuoka
**Year**										
2011	34.3	37.1	37.6	39.5	34.9	39.3	36.3	39.6	35.8	36.3
2012	34.7	35.0	36.2	38.3	35.6	40.2	35.3	39.9	36.6	33.6
2013	31.0	32.8	33.9	34.8	31.9	35.0	32.7	36.7	33.4	32.2
2014	30.6	34.6	33.2	35.3	32.0	36.7	34.8	36.8	33.6	32.2
2015	31.5	34.0	33.4	35.0	31.9	37.1	35.3	37.8	35.2	31.4
2016	30.2	31.8	32.0	33.6	33.0	37.9	33.7	37.4	35.9	31.0
2017	26.4	26.6	29.1	30.4	28.7	34.5	29.7	33.6	32.7	27.7
2018	22.2	23.4	27.2	26.6	25.4	30.7	25.5	30.6	29.8	24.9
2019	21.4	21.2	25.9	25.3	23.5	30.4	23.3	29.3	29.9	24.3
2020	13.8	13.3	16.1	16.6	15.3	18.4	15.1	18.5	18.9	15.5
2021	13.7	13.9	16.2	16.8	16.1	19.9	15.5	18.7	20.4	16.1
2022	10.1	11.4	15.2	16.1	14.4	16.3	13.5	16.0	17.8	16.0
**%Reduction**	**70.4**	**69.4**	**59.5**	**59.1**	**58.8**	**58.6**	**62.8**	**59.5**	**50.4**	**55.9**

Supplemental Table 7. Prefecture-Specific Trends in Antibiotic Use from 2011 to 2022 (3/3).

Prefecture	Saga	Nagasaki	Kumamoto	Oita	Miyazaki	Kagoshima	Okinawa
**Year**							
2011	32.0	33.8	38.1	39.5	38.2	36.0	34.3
2012	31.0	31.5	37.9	40.0	37.7	35.4	30.8
2013	29.4	29.2	37.5	36.1	36.7	33.7	31.8
2014	29.6	29.6	36.7	35.2	35.8	33.1	30.8
2015	30.1	28.9	36.1	36.3	38.5	33.6	29.7
2016	30.8	28.7	35.6	37.1	39.3	33.2	29.8
2017	27.8	26.2	31.1	32.6	34.3	29.2	27.5
2018	25.4	22.3	27.2	29.6	31.8	27.6	25.8
2019	25.0	21.8	26.4	29.9	31.2	27.0	25.8
2020	19.2	15.0	17.9	18.7	23.2	19.8	17.5
2021	19.2	14.8	16.9	19.4	22.9	18.9	17.1
2022	18.2	13.7	16.5	17.5	21.2	17.1	18.5
**%Reduction**	**43.0**	**59.5**	**56.8**	**55.7**	**44.6**	**52.6**	**46.1**

Supplemental Table 8. Interrupted Time-Series Analyses of Oral Antibiotic Use and Access Rate, 2011–2022.

**Interrupted time-series analyses**	**Oral antibiotic use model**				**Access rate model**			
	Coef.	Exp(Coef.)	95%LL	95%UL	Coef.	Exp(Coef.)	95%LL	95%UL
Baseline annual trend before 2014	β_1	0.951	0.920	0.984	γ_1	1.039	1.025	1.052
Changes in trend after 2014	β_2	1.057	0.999	1.118		N/A		
Changes in trend after 2016	β_3	0.918	0.883	0.954	γ_3	1.055	1.030	1.079
Changes in trend after 2020	β_4	1.076	1.048	1.105	γ_4	0.971	0.931	1.013
Immediate level change after 2020	β_5	0.636	0.602	0.674	γ_5	0.845	0.769	0.928
Estimated Slope								
2011–2014	β_1	0.951	0.92	0.984	γ_1	1.039	1.025	1.052
2015–2016	$\beta_1 + \beta_2$	1.006	0.977	1.036		N/A		
2016–2019	$\beta_1 + \beta_2 + \beta_3$	0.923	0.911	0.936	$\gamma_1 + \gamma_3$	1.095	1.081	1.110
2020–2022	$\beta_1 + \beta_2 + \beta_3 + \beta_4$	0.993	0.971	1.017	$\gamma_1 + \gamma_3 + \gamma_4$	1.063	1.022	1.107

*Note: Statistical models

1) Oral antibiotic use

$$\log(Y) = \beta_0 + \beta_1 T + \beta_2 T_{after\_2014} + \beta_3 T_{after\_2016} + \beta_4 T_{after\_2020} + \beta_5 I(T_{after_{2020}}) + \log(1000\ inhabitant - days)$$

where Y represents the days of therapy of oral antibiotic use, T denotes the number of years since 2011, T_{after_2014}, T_{after_2016}, and T_{after_2020} indicate the time elapsed after each respective intervention year, $I(T_{after_{2020}})$is an indicator variable equal to 1 for years after 2020 and 0 otherwise, and log(1000 inhabitant-days) is included as an offset term.

2) Access rate

$$\log(Y_{access}) = \gamma_0 + \gamma_1 T + \gamma_3 T_{after\_2016} + \gamma_4 T_{after\_2020} + \gamma_5 I(T_{after_{2020}}) + \log(Y_{total})$$

where Y_{access} represents the days of therapy for Access antibiotics, T denotes the number of years since 2011, T_{after_2016} and T_{after_2020} indicate the time elapsed after each respective intervention year, I(T_after2020) is an indicator variable equal to 1 for years after 2020 and 0 otherwise, and $\log(Y_{total})$ is included as an offset term to account for the days of therapy for total antibiotic use.

Supplemental Figure 1. National trends in antibiotic use from 2011 to 2022 stratified by antibiotic types.

Penicillin

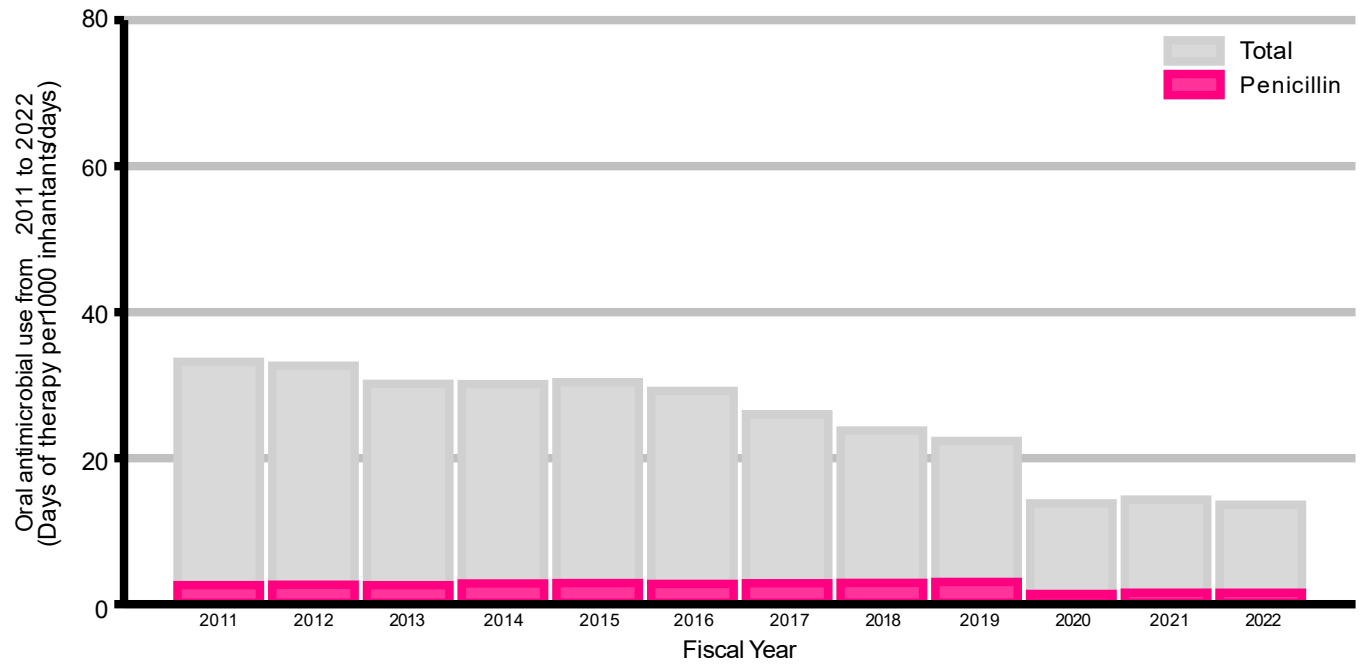

Penicillin + Beta-lactamase inhibitors

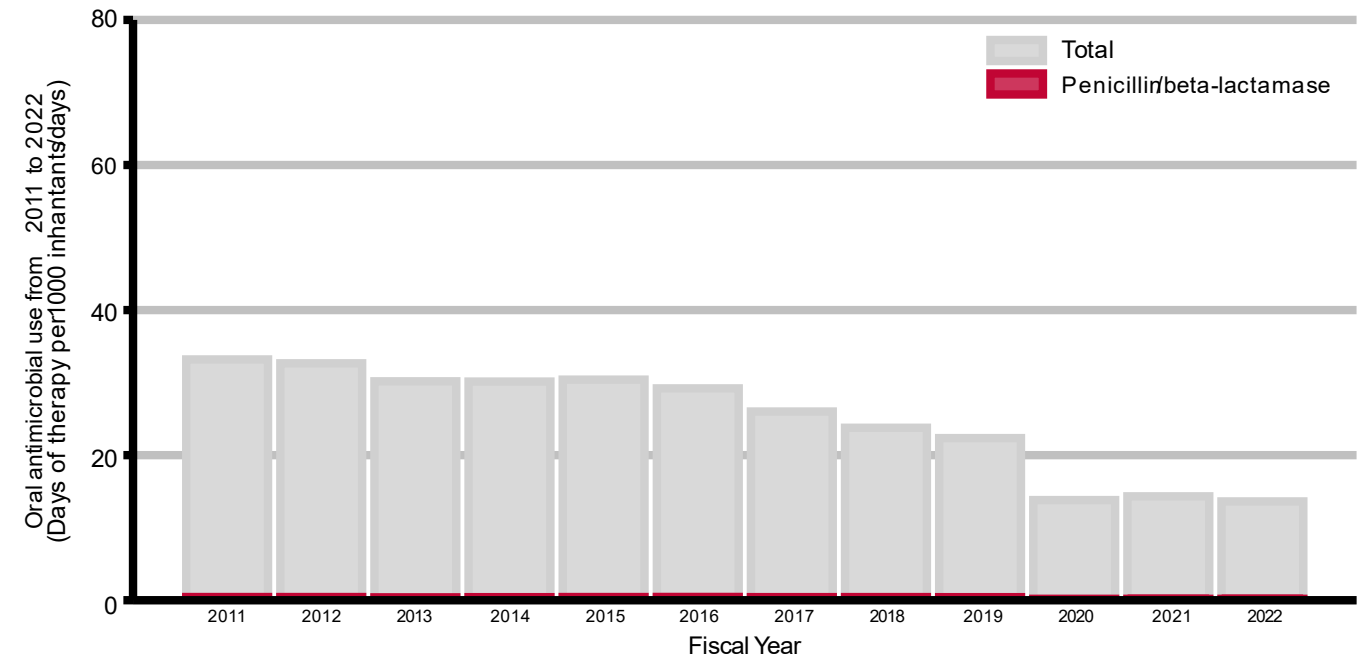

1st-generation cephalosporins

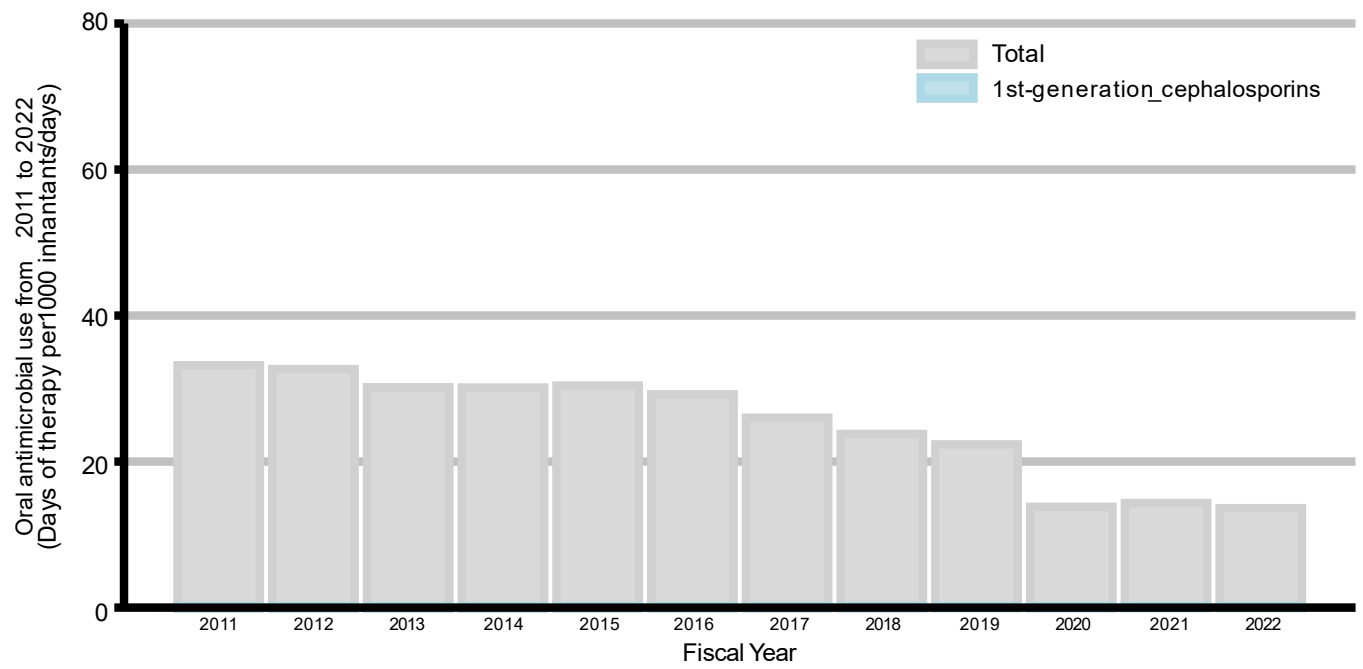

2nd-generation cephalosporins

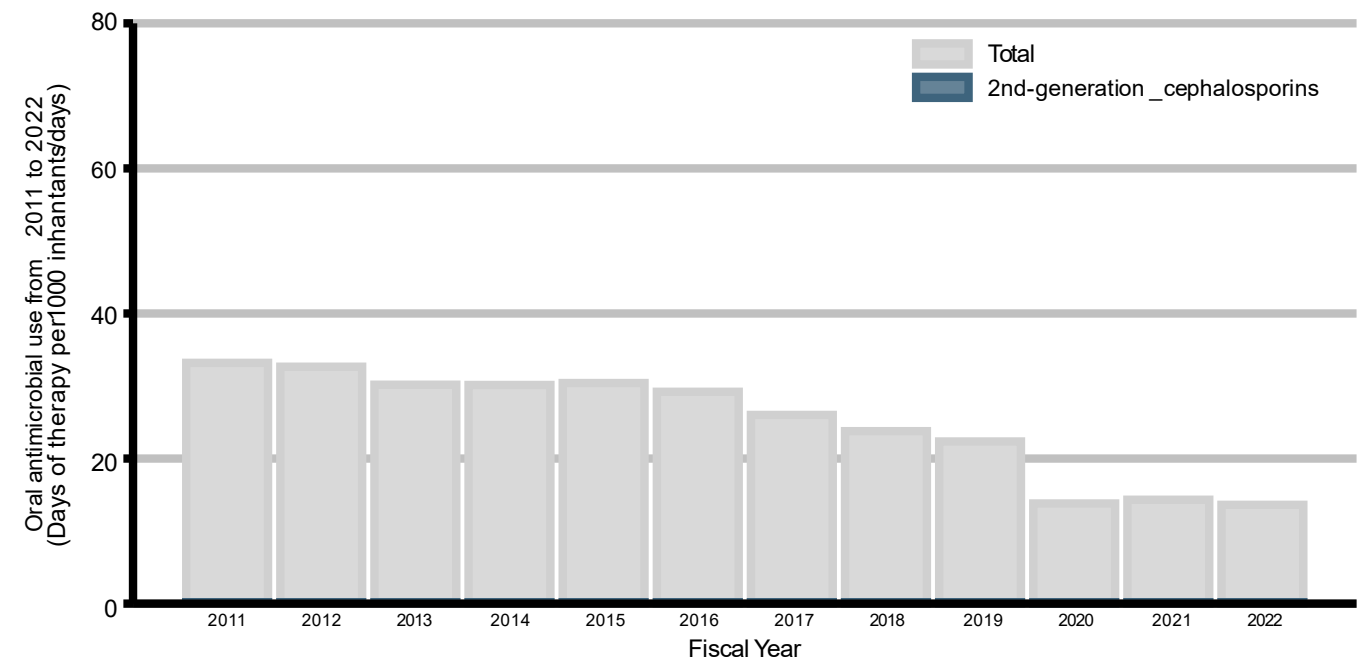

3rd-generation cephalosporins

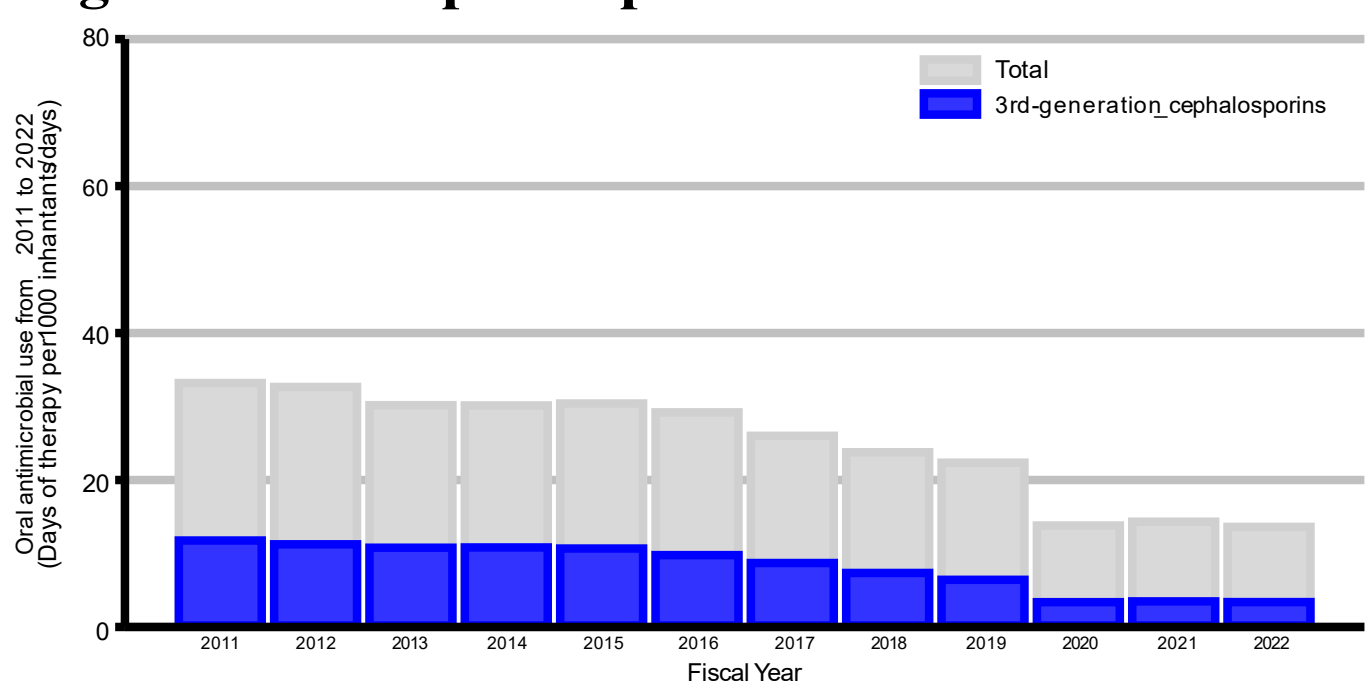

Oral penems

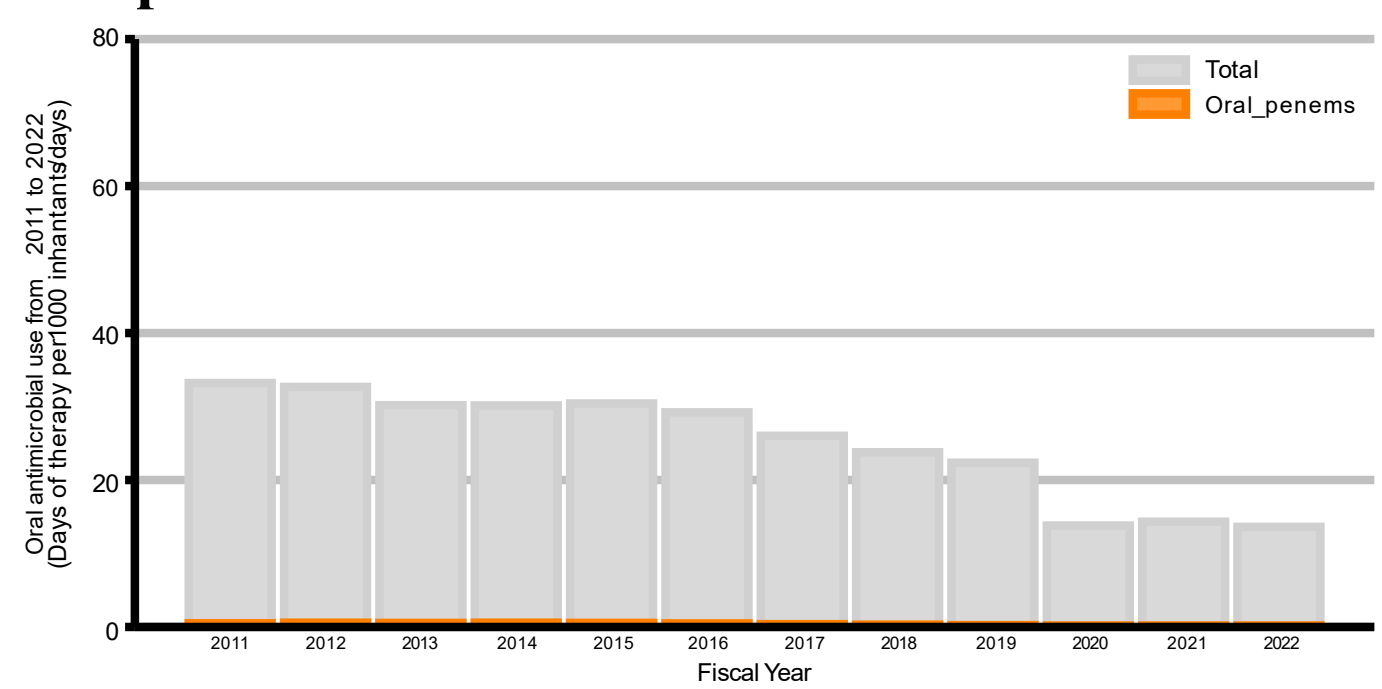

Macrolides

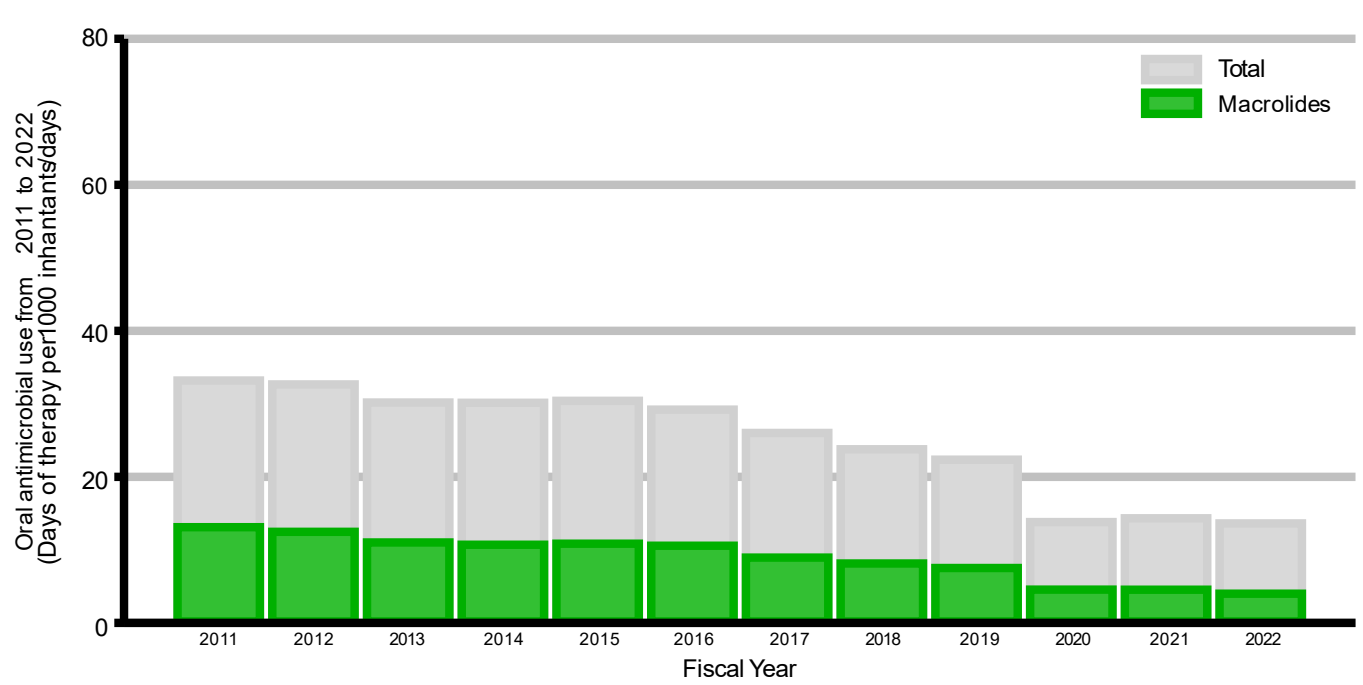

Tetracyclines

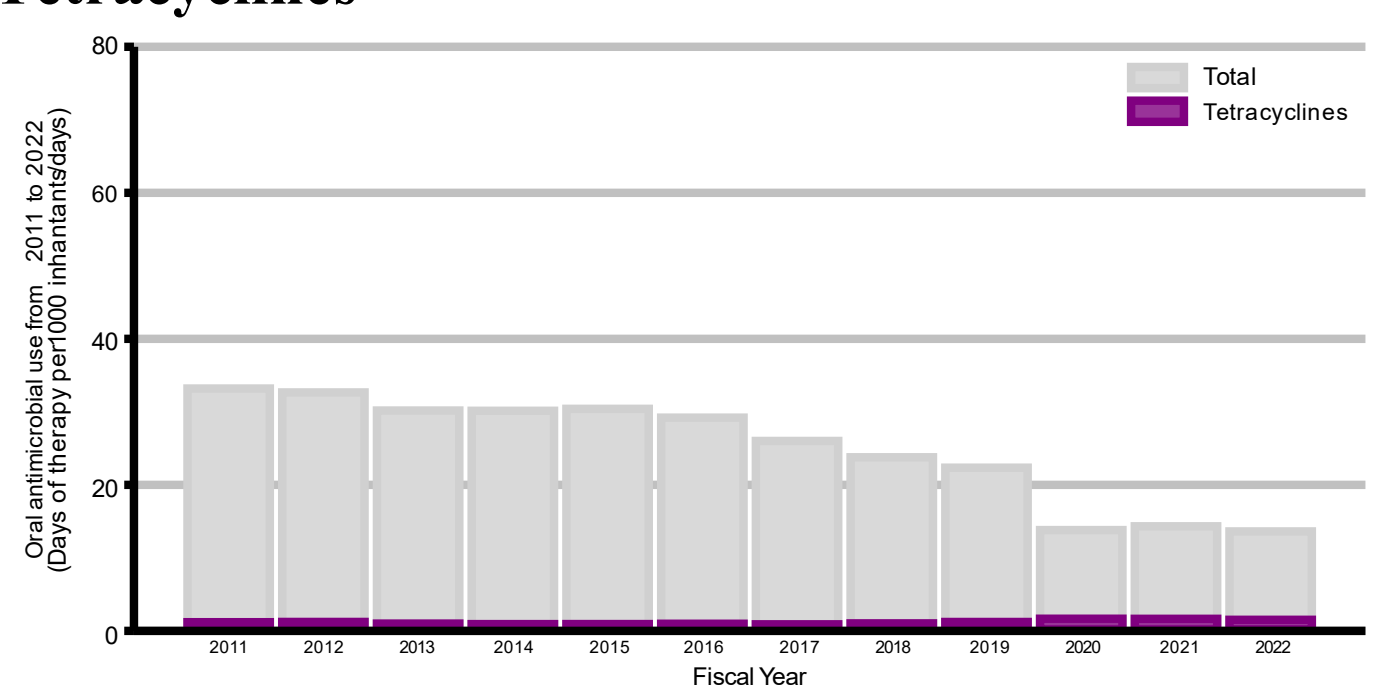

Quinolones

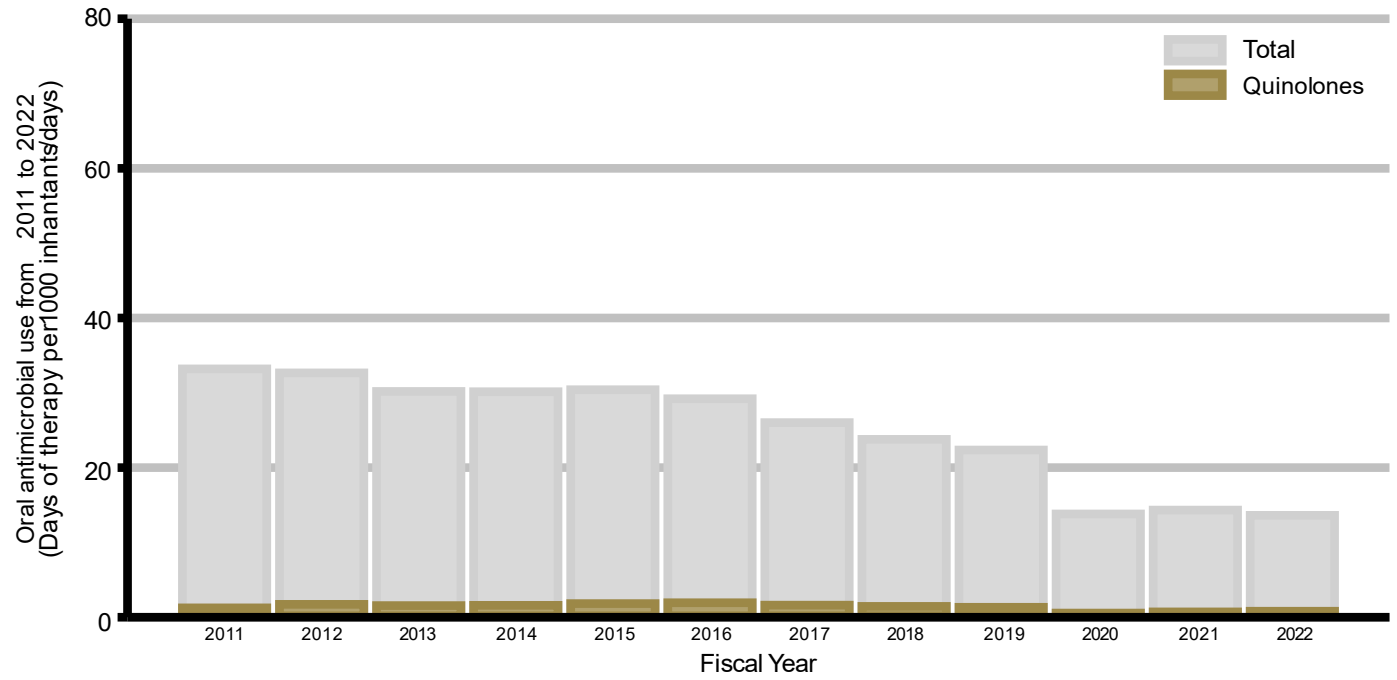

Sulfamethoxazole/Trimethoprim

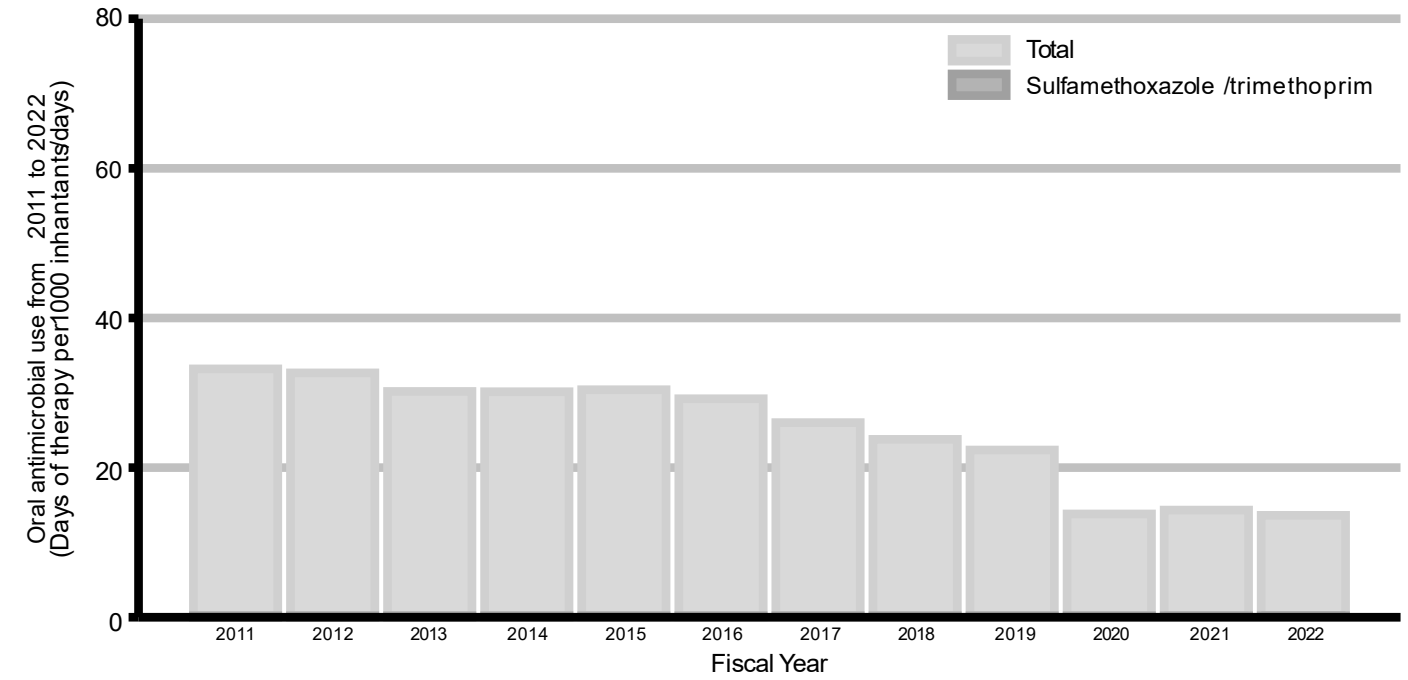

Others

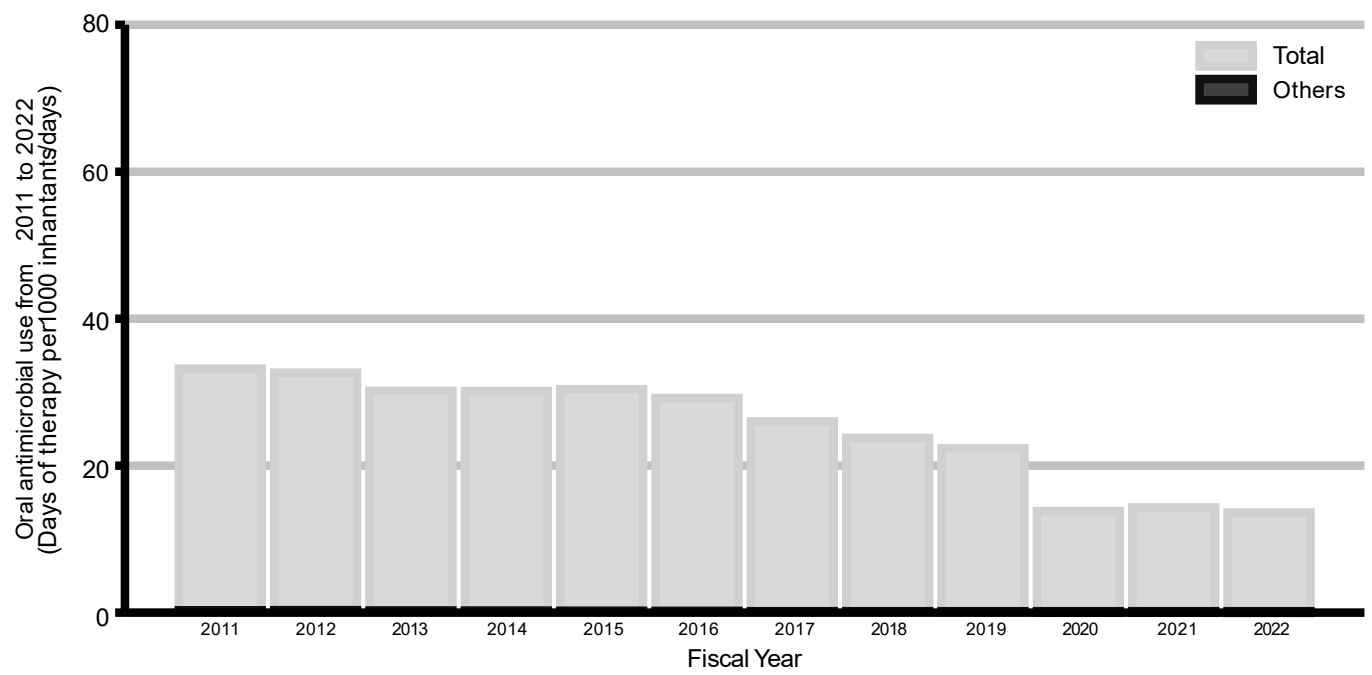

Total

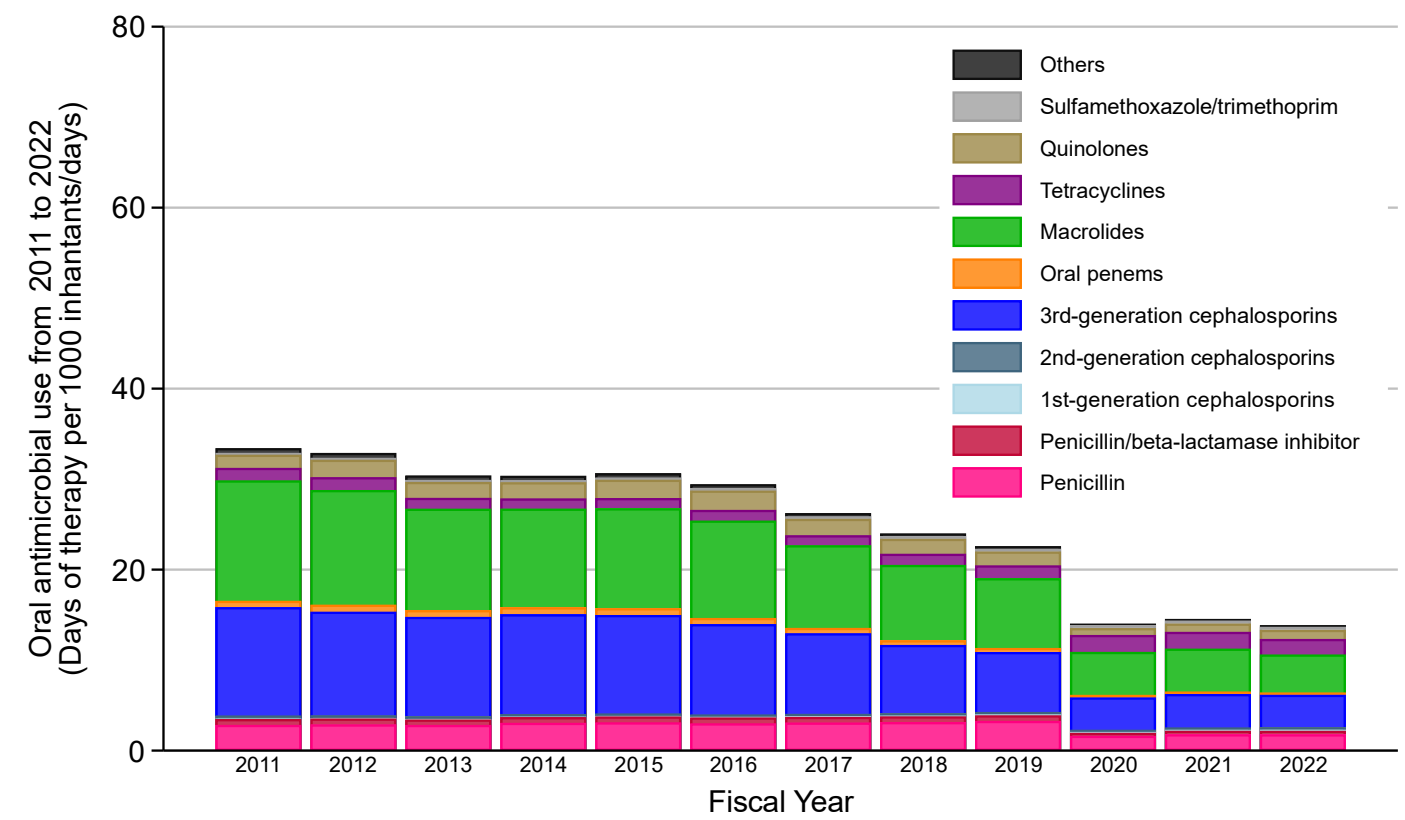

Supplemental Figure 2. National trends in access rates from 2011 to 2022.

Overall trends

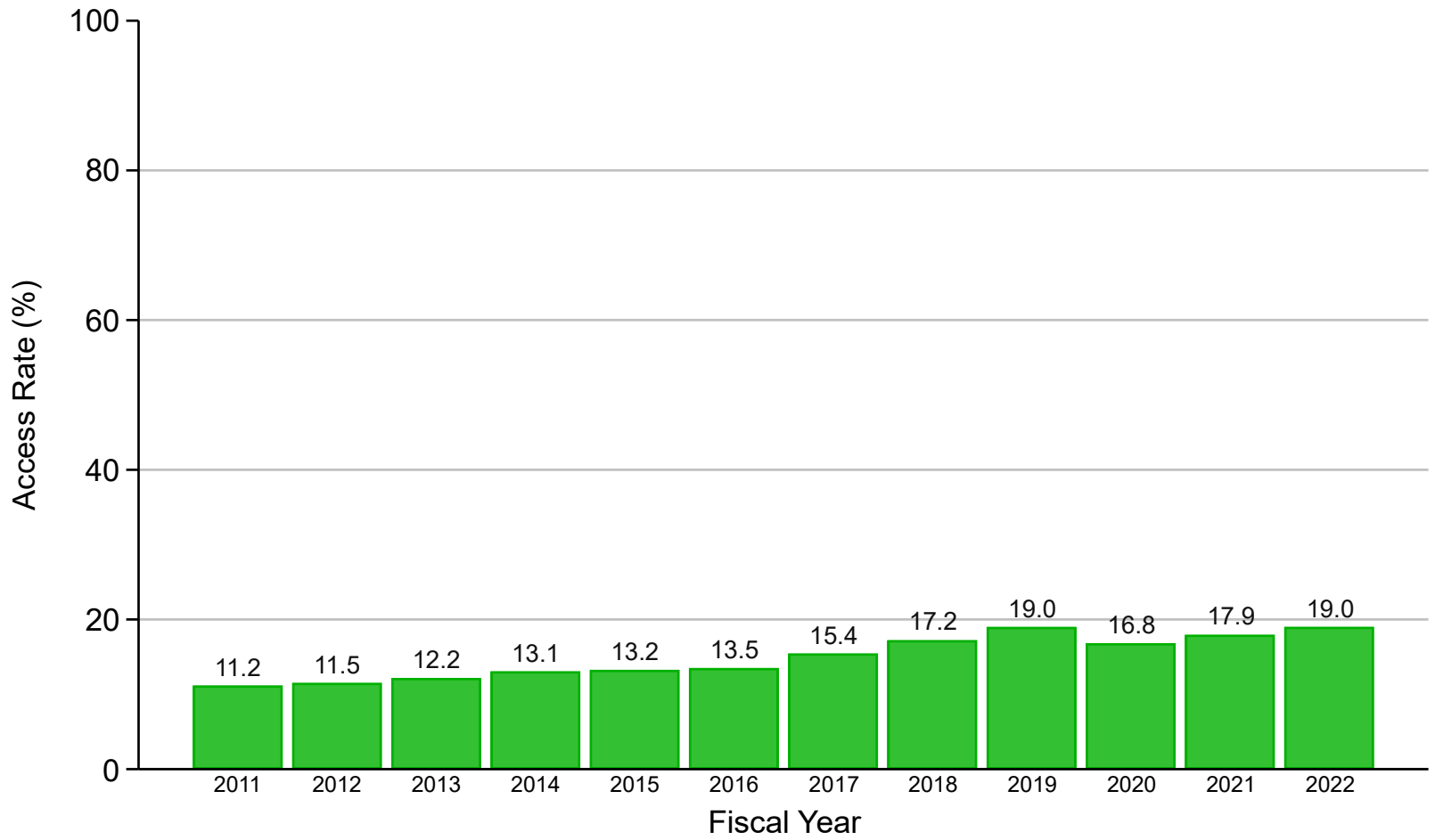

Trends by age group

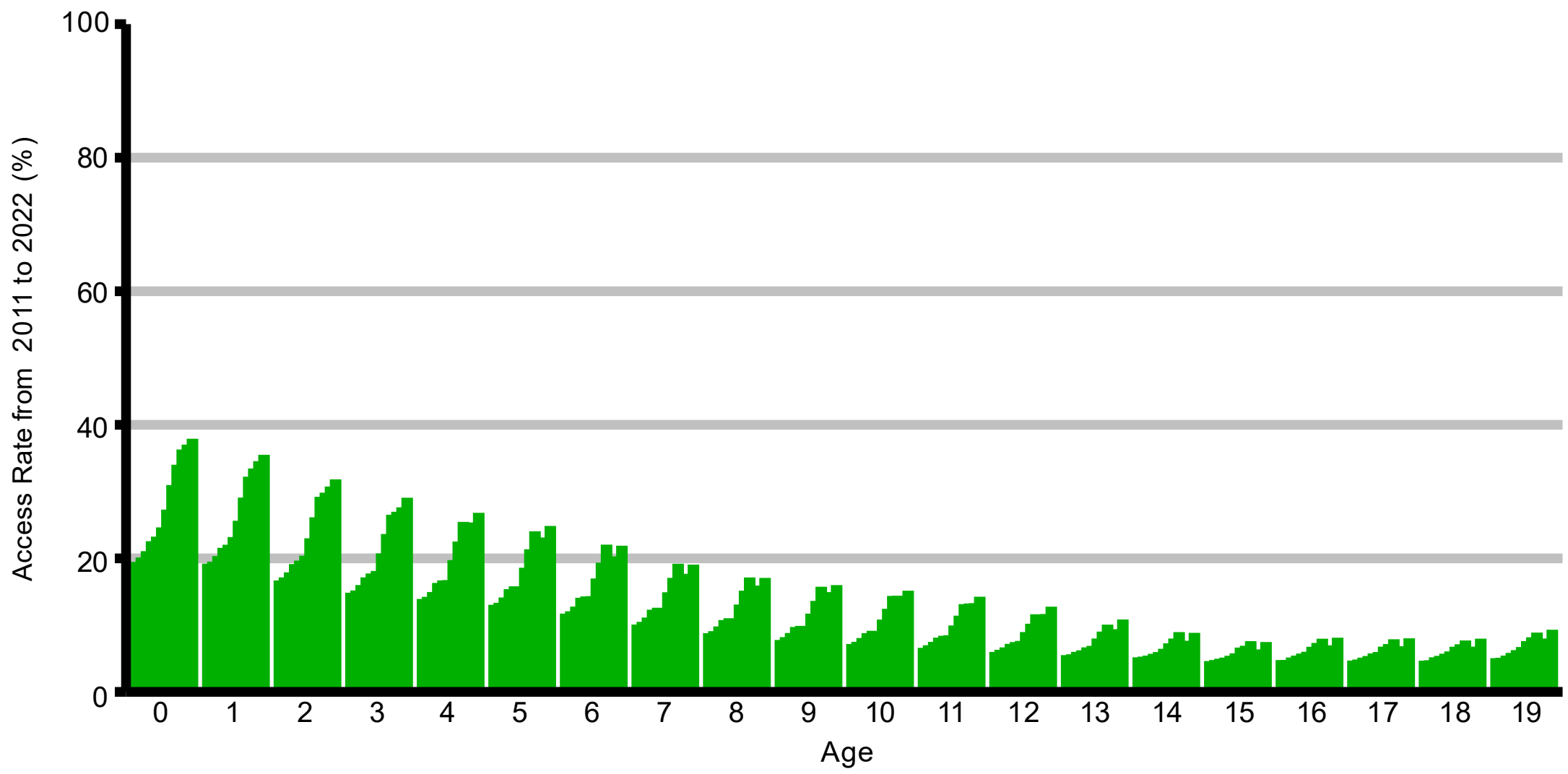

Trends by 47 prefectures

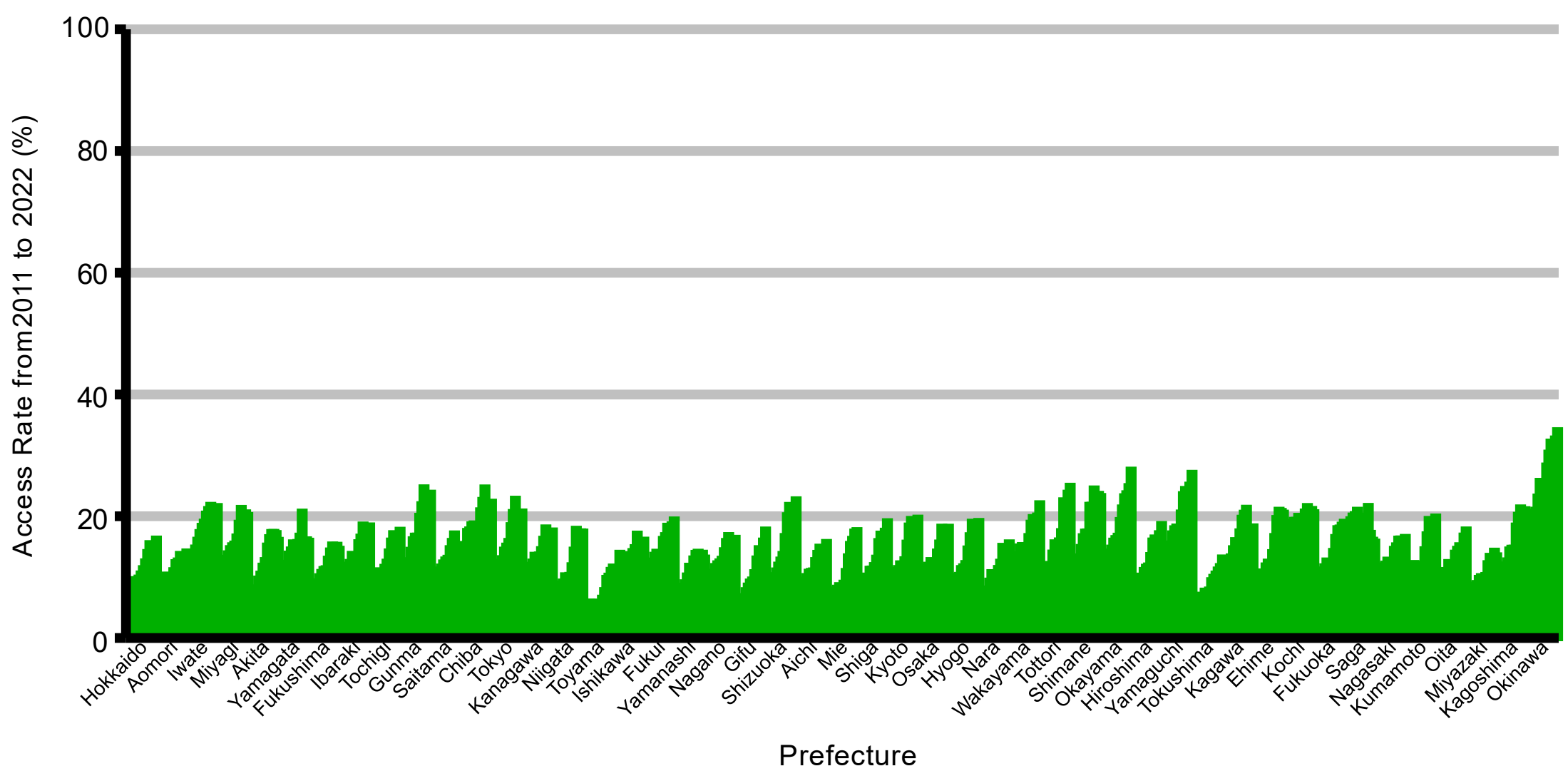

Supplement: Supplementary file 1 [file 2433-3298-9-5-1156-s001.pdf]
